# Telonemia-specific environmental 18S rDNA PCR reveals unknown diversity and multiple marine-freshwater colonizations

**DOI:** 10.1186/1471-2180-10-168

**Published:** 2010-06-09

**Authors:** Jon Bråte, Dag Klaveness, Tellef Rygh, Kjetill S Jakobsen, Kamran Shalchian-Tabrizi

**Affiliations:** 1 University of Oslo, Department of Biology, Microbial Evolution Research Group (MERG), N-0316 Oslo, Norway; 2 University of Oslo, Department of Biology, Centre for Ecological and Evolutionary Synthesis (CEES), N-0316 Oslo, Norway

## Abstract

**Background:**

Recent surveys of eukaryote 18S rDNA diversity in marine habitats have uncovered worldwide distribution of the heterotrophic eukaryote phylum Telonemia. Here we investigate the diversity and geographic distribution of Telonemia sequences by in-depth sequencing of several new 18S rDNA clone libraries from both marine and freshwater sites by using a Telonemia-specific PCR strategy.

**Results:**

In contrast to earlier studies that have employed eukaryote-wide PCR design, we identified a large and unknown diversity of phylotypes and the first rigorous evidence for several freshwater species, altogether comprising 91 unique sequences. Phylogenies of these and publicly available sequences showed 20 statistically supported sub-clades as well as several solitary phylotypes with no clear phylogenetic affiliation. Most of these sub-clades were composed of phylotypes from different geographic regions.

**Conclusions:**

By using specific PCR primers we reveal a much larger diversity of Telonemia from environmental samples than previously uncovered by eukaryote-wide primers. The new data substantially diminish the geographic structuring of clades identified in earlier studies. Nevertheless, since these clades comprise several distinct phylotypes we cannot exclude endemicity at species level. We identified two freshwater clades and a few solitary phylotypes, implying that Telonemia have colonized freshwater habitats and adapted to the different environmental and ecological conditions at independent occasions.

## Background

Microorganisms usually exist in populations of huge sizes and are highly prone to long-distance dispersal by vectors such as wind, water, animals and humans [1-5]. Obvious barriers to dispersal are lacking, especially in the marine habitat [4-8]. The ubiquitous dispersal of microorganisms has been a prevalent view since the turn of the last century, summarized in the statement "everything is everywhere, but, the environment selects" [9,10]. This view has been challenged however, by investigations of environmental DNA clone libraries as a large number of cryptic species and restricted biogeographies have been revealed [11-20]. High levels of genetic diversity have been found, even within the slowly evolving small ribosomal subunit gene [21,22]. However, as more localities are being investigated and the variety of sampling strategies increase, the geographic ranges of many microorganisms have been expanded, showing that under-sampling of the diversity can cause a false impression of endemism [see [4,5]]. Some surveys have therefore interpreted the diversity as consistent with the "Moderate Endemicity Model" (MEM), which states that some microbial lineages do in fact have a global distribution, but that there also exists species with restricted dispersal and local adaptations [4,23-25].

The vast majority of 18S rDNA environmental surveys conducted so far have involved universal primers designed to capture the broadest diversity of eukaryotes possible. However, much diversity is most likely overlooked by applying only a single pair of universal primers [26-28]. This could be due to a number of reasons, e.g. the primers are less suitable for some groups of organisms, there are great variations in rDNA copy number, as well as bias introduced in the PCR reaction. One of the most efficient approaches to address these problems has been to apply a group-specific PCR strategy with primers targeting the particular taxonomic group of interest [29-32]. These studies have shown that the use of such primers is detecting far more diversity than the universal approach.

Telonemia is one of the groups of unicellular eukaryotes that are frequently detected in marine 18S rDNA environmental clone libraries, but usually represents only a relatively small part of the total diversity [11,33-36]. So far, only two species have been described on the basis of morphology, *Telonema subtilis *Griessmann and *T. antarcticum *Thomsen *in *Klaveness et al. [20,37], but the size range of the identified species is large (3.5 - 15 μm long and 4-20 μm wide). It was recently discovered by Shalchian-Tabrizi et al. [36] that the 18S rDNA sequences formed two major groups, Group 1 and 2, including *T. subtilis *and *T. antarcticum *respectively, and that these were further sub-divided into several statistically supported clades of sequences with restricted geographic distribution.

Species of Telonemia are heterotrophic predators, feeding on a wide range of bacteria and pico- to nano-sized phytoplankton. They are globally distributed in marine waters and are frequently encountered in environmental clone libraries e.g. [34,38]. Telonemia are present throughout the year and are considered to play an important ecological role, as they have been found to dominate the heterotrophic protist community on certain occasions [37]. Very little is known about the life cycle and reproduction of Telonemia. Asexual reproduction occurs by cell division and the possible presence of cysts has been indicated by Vørs [39], but this is yet to be verified.

Telonemia has also been reported from fresh water habitats. Tong et al. [40] identified a freshwater *T. subtilis *in an Antarctic lake, Sombre Lake, but it is unclear if this specimen is truly freshwater as the lake has been classified as maritime [41]. A survey of Finnish lakes recorded *Telonema *sp. on a number of occasions (Liisa Lepistö, personal communication). The ability to survive under low salinity conditions have also been shown in culture experiments done on *T. subtilis *from Norwegian coastal waters [42]. Although Telonemia has been observed at several occasions in freshwater, only a few 18S rDNA sequences appear to be related to the group [43]. Therefore, it is still unclear how large the diversity of Telonemia might be in these habitats and what phylogenetic relationship they have to marine species. It is also unclear whether Telonemia have colonized these habitats at one or several independent occasions, and if both the two major groups related to *T. subtilis *and *T. antarcticum *have been successfully established in freshwater.

Here, we have designed Telonemia-specific 18S rDNA primers in order to investigate (i) whether group-specific environmental PCR will uncover a larger diversity of Telonemia than so far uncovered by universal primers, (ii) whether increased taxon sampling will affect the geographic structuring observed for many clades of marine Telonemia [36], and (iii) to examine whether one or several species exist in freshwater, and whether both Group 1 and 2 comprise species from freshwater. We address these questions by sequencing clone libraries from 4 marine and 3 freshwater localities, as well as including all available Telonemia sequences already published. We identify a large and unknown diversity of phylogenetically distinct sequences (phylotypes) in marine waters and provide the first rigorous evidence for the presence of several freshwater species related to both *T. subtilis *and *T. antarcticum *resulting from independent colonisations of freshwater.

## Results and discussion

### Large cryptic diversity of Telonemia in marine habitats

Despite the huge amount of environmental 18S rDNA sequences from numerous diversity studies available in public databases, only 33 were found to belong to Telonemia in Shalchian-Tabrizi et al. [36], all amplified by universal eukaryotic primers. These sequences were divided into two main groups, Group 1 and Group 2, including *T. subtilis *and *T. antarcticum *respectively [36]. Within these groups, twelve distinct sub-groups or independent phylotypes were identified, each possibly representing several species or populations. The majority of these clades were composed of sequences from single localities, suggesting a considerable geographic structuring of Telonemia [36].

By using group-specific primers we generated 145 18S rDNA sequences affiliated to Telonemia. No sequences from other eukaryote groups were generated. The evolutionary origin of these sequences was inferred by phylogenetic analyses of an alignment containing a broad diversity of eukaryotic lineages (alignment 1) that included our new data and all putative Telonemia sequences downloaded from GenBank (result not shown). Hence, the group specific PCR strategy for Telonemia clearly improves our knowledge about the diversity of the group.

**Figure 1:**
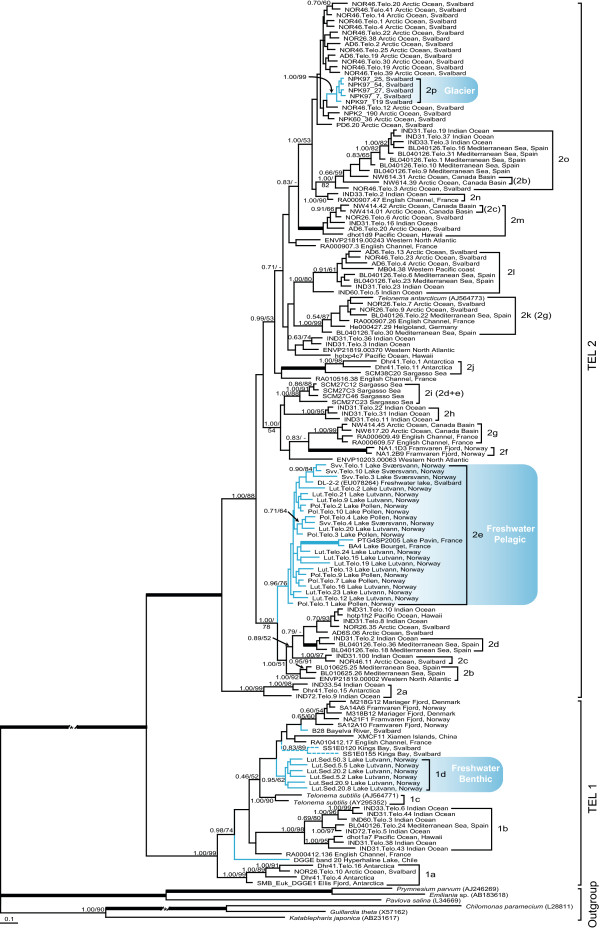
**Bayesian phylogeny showing the relationship of the Telonemia 18S rDNA sequences**. Numbers at the nodes represent Bayesian and Maximum Likelihood support values respectively. Names in brackets indicate sub-groups recognized in [36] that are referred to in the text. Only values above 50/0.70 are shown and thick branches indicate full statistical support (100/1.00). Blue lines show freshwater sequences and dashed blue lines indicate possible freshwater origin. An asterisk (*) indicates that branch length has been cut in half.

To better resolve the phylogeny of the Telonemia sequences we removed all other eukaryote groups (except haptophytes, cryptophytes and katablepharids used as outgroups) that allowed for inclusion of more unambiguously aligned nucleotide characters (i.e. alignment 2). This phylogeny recovered Group 1 and 2, here renamed to TEL 1 and TEL 2 respectively, with high support (1.00 posterior probability (pp) and >99% bootstrap support (%); Figure 1). Furthermore at least 20 sub-groups (1a-1d and 2a-2p in Figure 1) were supported with substantial statistical support. Several of these groups could perhaps be even further subdivided, based on the internal support values (e.g. groups 1b and 2i) but are here treated as single groups for simplicity. The naming of the groups follows that of Shalchian-Tabrizi et al. [36] and has been extended here to include the new sub-groups.

As previously recognized, TEL 1 contained fewer clades than TEL 2 and is here divided into 4 sub-groups. Earlier, TEL 1 was found to be restricted to the English Channel and Danish waters [36], but is here extended to include phylotypes from the Antarctic and Arctic regions as well as warmer waters such as the Indian Ocean, Hawaiian waters, the Mediterranean Sea and Chinese waters. In TEL 2 we could now identify 16 sub-groups sampled from worldwide locations such as the Arctic and Antarctic oceans, the English Channel, Danish and German waters, the Indian Ocean, Sargasso Sea, Mediterranean Sea and Hawaiian waters.

### Implications on the geographic structuring of Telonemia

The geographic structuring shown by Shalchian-Tabrizi et al. [36] is here diminished by the addition of more environmental sequences (Figure 1). Several of the sub-groups previously found to have restricted geographic ranges now includes sequences from new locations. For instance the sub-groups 2m and 2o (earlier 2c and 2b; Figure 1), previously found to be restricted to the Arctic Ocean, are now extended to the Indian Ocean, Hawaiian waters and the Mediterranean Sea. The sub-group 2k (earlier 2g), which was previously restricted to the English Channel, Oslo Fjord and Helgoland (i.e. southern parts of the North Sea/Skagerak), now includes sequences from the Mediterranean Sea as well. Additionally, most of the sub-groups new to this study have widespread distributions; e.g. sub-group 1b is composed of sequences from the Indian Ocean, the Mediterranean Sea and the Pacific Ocean, while sub-group 2l is composed of sequences from as distant locations as the Arctic Ocean, Western Pacific, Mediterranean Sea and the Indian Ocean. Although the majority of the subgroups show little geographic structuring, the high diversity uncovered here implies that geographical isolation has existed at some point. The combination of high diversity and low geographic structuring show that subsequent dispersal rates have been higher than speciation rates over the history of Telonemia.

The existence of endemicity cannot be completely excluded however. One important reason is that each clade may represent higher order taxonomic units, like genera or families, and each phylotype can in principle represent separate species (or even several species as 18S rDNA may be too conserved to demarcate species boundaries [21,25,44]). Hence, the widespread geography of the subgroups may be hiding endemicity at strain or species level; in fact we could not identify the same phylotypes from different localities. Sampling of DNA from more sites and a larger variety of marine habitats, as well as the use of faster evolving genetic markers, such as the internal transcribed spacer (ITS) of the ribosomal operon, would be necessary to resolve this question. On the other hand, any putative geographic restriction of species or groups should be interpreted with caution because endemicity in general is difficult to prove, as there will always be a possibility of undersampling and absence of species at times of sampling due to seasonal variations.

There are a few tendencies of endemicity or restricted biogeography in the inferred phylogeny (Figure 1) that deserve to be noted here and should be subject for future studies of Telonemia. In particular, a striking pattern is seen for sequences from Antarctic and Arctic regions clustering into sub-group 1a, which opens up the possibility for a bi-polar or anti-tropical distribution. If further diversity studies confirm this pattern, it would be congruent with geographic distribution of dinoflagellates and foraminiferans [45,46]. Three other clades also appear to be endemic; the clade 2i from the Sargasso Sea and 2h and 2f, are only composed of Indian Ocean and the Norwegian Framvaren Fjord sequences respectively (Figure 1). In addition there is a large assembly of sequences from the Svalbard region that could indicate the presence of a Norwegian-Barents Sea population, but this assembly is only moderately supported (Figure 1).

### Cryptic diversity of Telonemia in freshwater

In order to investigate the putative existence of Telonemia in freshwater we had to use a nested PCR amplification strategy. This could explain why so little sequence data from Telonemia in freshwater has been generated previously and confirm visual observations that freshwater Telonemia exists only in minute quantities (L. Lepistö unpublished). The sequences obtained from the three different Norwegian freshwater lakes, Lake Lutvann, Lake Sværsvann and Lake Pollen, together with a few publicly available freshwater environmental sequences, formed three clades (1d, 2e and 2p) and two single phylotypes with representatives in both TEL 1 and TEL 2 (Figure 1). In Lake Lutvann we sampled both the sediment and the water column. Strikingly, these sequences formed two distantly related habitat-specific clades, in which all the benthic sequences clustered into one group (1d) and the pelagic sequences into another (2e), highlighting a vertical stratification of phylotypes or populations within this lake at the time of sampling (Figure 1). Sub-group 2e was in addition composed of sequences from the pelagic zone of the two other Norwegian lakes as well as three other freshwater sequences from Svalbard and France.

A few other phylotypes in TEL 1 may represent additional successful transitions from marine to freshwater lakes. One sequence (DGGE band 20) is sampled from a hyperhaline lake in Chile, Lake Tebenquiche that is situated in the Andes at 2500 m.a.s.l. The lake is classified as hyperhaline but has extreme variations in salinity, ranging from 1% to 30% [47]; hence the potential Telonemia species from this lake could be adapted to any of these salinity conditions or could simply be a marine species that have dispersed into the lake. Another sequence (B-2-8), is sampled from the Bayelva River in Svalbard, which is composed of glacial melt water as well as water from nearby freshwater lakes [48], and discharges into the Kings Bay delta in Spitsbergen. Interestingly the two sequences (SS1E0120 and SS1E0155) that have been obtained from the Kings Bay is branching off close to the sequence from the Bayela River in Figure 1, and may therefore actually have freshwater origin.

A third cluster of freshwater sequences (2p), entirely composed of sequences sampled from a glacier in Svalbard, belonged to TEL 2. This cluster was distantly related to the other freshwater group (2e) and was embedded in a large assembly of Arctic and Antarctic sequences, although this relationship was weakly supported (Figure 1). *T. subtilis *is commonly observed inhabiting the sea-ice in the Baltic Sea [49] and it is therefore possible that these sequences originate from a marine species transported onto the glacier from marine waters by aerosols or other vectors. On the other hand, if these represent an actual freshwater species this would be a second freshwater species within TEL 2, distantly related to the Bayelva River sequences. It remains to be verified that these are actually living cells and whether these have been transported from freshwater sources or dispersed on to the glacier from marine habitats via aerosols or other vectors. So far, we have not detected sequences from the marine samples that are identical to these glacier phylotypes, which could indicate such freshwater dispersal, but as only few samples have been made in these areas we cannot exclude this possibility.

### Few marine-freshwater cross-colonizations

In Figure 1 the freshwater sequences form distinct clusters and phylotypes, suggesting the existence of several different freshwater species. These are placed within both TEL 1 and TEL 2, demonstrating that relatively distantly related species of Telonemia exists in freshwater. This diversity is detected even with a very limited number of samples; we therefore expect future surveys of other types of freshwaters at other continents to uncover an even larger diversity. The clustering pattern of the Telonemia sequences is in accordance with recent studies of other protist groups showing that freshwater species form distinct clades in phylogenetic trees, i.e. they are more closely related to each other than to marine species [reviewed in [50]]. Such clustering pattern of freshwater phylotypes has in these studies been interpreted as successful marine-freshwater transitions. These transitions have often been ancient and rare events, resulting in most of the extant species being restricted to either of the two habitats: e.g. in bodonids [51], goniomonas [52], cryptomonads [53], dinoflagellates [54] and Perkinsea [55]. If further examinations of freshwater with the use of Telonemia-specific PCR approaches confirms the clustering pattern shown here (see Figure 1), it would imply that the biogeophysical differences between marine and fresh waters constitutes a significant ecological barrier for dispersal of Telonemia that affects diversification of the lineage. In addition to more samples from various freshwater localities, a better identification of the species is necessary in order to further reveal the species diversity. For instance, analyzing RNA to confirm that the species are alive and metabolize in the habitat, and fluorescence in situ hybridization (FISH) would be helpful in relating the sequences to the actual cells in the habitats, and to better understand whether the dispersal of species between different and similar habitats take place in the form of spores or as active cells.

Each of the freshwater clades in our tree are habitat-specific in that they only contain phylotypes from either sediment (clade 1d), pelagic (clade 2e), and potentially also glacier (clade 2p) and hyperhaline habitats, implying that each of these habitats have possibly been colonized independently by marine species and adapted to different environmental and ecological conditions. Interestingly, this clustering pattern indicate the existence of ecological barriers also between freshwaters habitats, but as this study primarily has focused on revealing the existence of Telonemia in freshwater, the geographic distribution of the various strains and species should be addressed by much more extensive sampling and adequate molecular methods.

## Conclusions

Here we have applied a group-specific PCR approach to better understand the diversity of Telonemia and to investigate whether the geographic structuring observed in earlier studies has been affected by undersampling. Our results show that the use of group-specific primers will uncover a much larger diversity from environmental samples compared to eukaryote-wide primers. The Telonemia-specific primers and the PCR protocol presented here were highly specific for the Telonemia group as no sequences from other eukaryote groups were identified in our sequence libraries. Further, the geographic structuring of marine groups found in earlier analyses is clearly diminished by the addition of the newly generated sequences, showing that undersampling of the diversity may lead to a false impression of endemicity. However, as only two species of Telonemia are defined on basis of morphology, it is not clear what taxonomic units the identified clades represent. Most likely each of these sub-groups are composed of many distinct species, as they comprise phylotypes with different 18S rDNA sequences. If each phylotype is representing separate species, it will be a tremendous task to understand the geographic distribution of each. Nevertheless, congruent with other recent studies [29-32] we have clearly shown the importance of using a group-specific PCR approach to better understand the cryptic diversity of protist groups. Studies of endemicity could be further undertaken by designing procedures that target each of the subgroups detected here and complemented with FISH and RNA sequencing strategies to verify that the species actually inhabit the location. For species or population demarcation, other faster evolving markers, such as ITS, may be needed. There is however a challenge when studying uncultured species to directly compare the phylogenetic clades based on different genes.

We provide here the first rigorous evidence for the existence of freshwater Telonemia. Two groups of freshwater sequences are identified showing that multiple and independent transitions from marine to freshwater have taken place during the evolutionary history of the group. It is obvious that the diversity of freshwater Telonemia is highly underestimated, and the ecological roles of Telonemia in these habitats are so far very much unclear. The possible stratification of species in freshwater is a first glimpse of potential differences in ecological adaptations - more studies combining molecular and microscopy approaches are clearly necessary to assess the diversity and dispersal patterns of Telonemia.

## Methods

### Environmental samples

Freshwater samples were collected from three different Norwegian lakes in May 2007; Lake Lutvann (59°54'N and 10°52'E) a small and deep (Zmax = 52 m) clearwater oligotrophic lake with long retention time, Lake Pollen (59°44'N and 10°45'E) a small and meromictic lake of intermediate depth (Zmax = 18 m) with only 7 m of freshwater and seawater in the monimolimnion, and Lake Sværsvann (59°48'N and 10°53'E) a small and shallow (Zmax = 11 m) meso- to polyhumic lake of complex morphology. Two litres of surface water (down to 50 cm) was collected from each lake and filtered through a Whatman GF/C glass-fiber filter with pore sizes of approximately 1 μm. Filters were dried and stored at -20°C. Sediment samples from Lake Lutvann were collected with a simple gravity corer at three depths, 50 m, 20 m and 5 m. The sediment samples from Lake Lutvann, including up to 500 ml of lake water were kept at 17°C with a 14/10 h light/dark cycle. 100 ml of culture of the cryptomonad species *Plagioselmis nannoplanctica *was added on average every three days for the Telonemia species to feed upon for seven days. *P. nannoplanktica *was grown in the freshwater media of Guillard & Lorenzen [56] without organic buffer.

Marine DNA was sampled from the following locations; Antarctica (59°22'S, 55°46W, December 1998), The Arctic Ocean (NOR26 and PD6 samples: 76°19'N, 23°45'E and NOR46 and AD6 samples: 76°20'N, 03°59'E, August 2002), The Mediterranean Sea (41°40'N, 2°48'E, January 2004) and the Indian Ocean (31°45'S, 52°37'E, May/June 1999). For sampling and DNA isolation methods see [11,57-59].

### DNA isolation and sequencing

DNA was isolated from the different freshwater samples by using the Power Max Soil DNA Isolation kit (MoBio, USA) following the manufacturers instructions. For DNA isolation from the sediments, 15 ml of sediment from the top layer were collected and centrifuged at 4000 rpm for 10 minutes. The isolated DNA was stored at -20°C.

**Figure 2:**
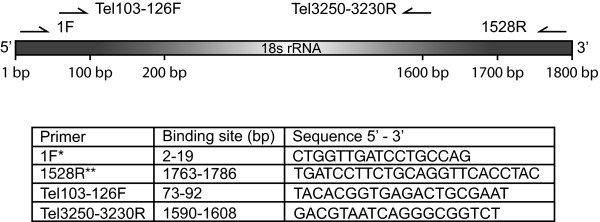
**Primers used in this study and their relative position in 18S rDNA gene**. * indicates that primer is based on PrimerA and ** indicates that primer is based on PrimerB designed by Medlin et al. [55]. The 18S rDNA gene in the figure is based on the *Telonema antarcticum *sequence AJ564773 (1787 bp) in GenBank [62].

Nested PCR was used to amplify the 18S rDNA gene from the freshwater samples with universal eukaryotic primers (based on PrimerA and PrimerB by Medlin et al. [60]; here called 1F and 1528R) used in the first round of amplification and Telonemia-specific primers used in the second round (for primer sequences, see Figure 2). The amplifications were done on an Eppendorf Mastercycler ep (Eppendorf, Germany) and a Biometra Thermocycler (Biometra, Germany) with a sample volume of 25 μl containing 10 - 200 ng of template DNA, 1 × HotMaster Taq Buffer with 2.5 mM Mg^2+ ^(5 Prime, USA), 200 μM dNTPs, 0.2 μM of each primer and 1.5 U HotMaster Taq DNA Polymerase (5 Prime, USA). The reaction mixture was incubated at 94°C for 2 min, followed by 30 - 34 cycles of 45 s at 94°C, 45 s at 60°C, 135 s at 72°C with a final extension at 72°C for 10 min. The PCR products were gel-extracted and purified using Wizard SV Gel and PCR Clean-Up System (Promega, USA), and cloned using TOPO TA Cloning Kit (Invitrogen, USA) following the manufacturers instructions. Colonies were checked for positive inserts by PCR amplification with the primers TopoF (5'-GGCTCGTATGTTGTGTGGAATTGT-3') and TopoR (5'-CCGTCGTTTTACAACGTCGTGACT-3') and identical reaction mixtures as described above, except that DynaZymeII (Finnzymes, Finland) DNA polymerase (1.5 U) and 1 × DynaZyme buffer (F-511) were used. The PCR program was as follows: Initial denaturation at 95°C for 5 min, 34 cycles of 15 s at 95°C, 30 s at 60°C, 120 s at 72°C with a final extension at 72°C for 7 min. The positive inserts were sequenced on an ABI 3730 DNA Analyzer (Applied Biosystems, USA) with the primers M13F and M13R (Invitrogen, USA) using the ABI BigDye terminator v3.1 kit (Applied Biosystems, USA). 183 clones were randomly picked from the generated libraries and sequenced with the M13F primer (Invitrogen, USA). Identical, or nearly identical, sequences were not sequenced further. 82 of the inserts were full-length sequenced (approximately 1500 bp) with the M13R primer (Invitrogen, USA). Accession numbers for sequences generated in this study [GenBank: GQ365764-GQ365903 and GU117661-GU117693].

### Phylogenetic analyses

Available sequences of possible Telonemia origin were identified by BLAST searches against the Entrez Nucleotide database [61,62] using sequences of known Telonemia origin as query. The sequences identified from the BLAST searches were downloaded and pooled into a local database together with the sequences generated in this study. These sequences were added to an 18S rDNA alignment of all the major eukaryotic groups (hereafter called alignment 1) to confirm relationship to Telonemia. After removal of ambiguously aligned characters using the program MacClade version 4.07 [63], alignment 1 consisted of 374 taxa and 1465 characters. Alignment 1 was subjected to maximum likelihood (ML) analyses by using the program RAxML v.6 [64]. The topology with the highest likelihood score out of 100 heuristic searches, each from a random starting tree, was selected, and bootstrapping was done with 100 pseudoreplicates and one heuristic search per replicate. In the ML analyses, the General Time Reversible (GTR) model, with a gamma-distributed rate of variation across sites (G), was employed.

The ML analyses of alignment 1 showed that 198 sequences grouped together within Telonemia (results not shown). To be able to include more unambiguously aligned characters, a second alignment (alignment 2) was created with MacClade version 4.07 [63], consisting of the Telonemia sequences identified in the analysis of alignment 1. Identical sequences were excluded and the putative closest sister groups of Telonemia, the cryptomonads, haptophytes and katablepharids, were used as an outgroup [20]. Chimeric sequences were identified as described in [65]. The sequence NW614.39 is chimeric with the last 100 bp from a diatom. This part of the sequence was not included in the analyses. Accession numbers and clone names of sequences in alignment 2 are given in Additional file 1. Alignment 2 consisted of 159 taxa and 1758 characters. This alignment was analysed by ML (as for alignment 1) and Bayesian inferences. The Bayesian inferences were done with the program MrBayes [66] as follows: two independent runs, each with three cold and one heated MCMC (Markov Chain Monte Carlo) chains were started from a random starting tree. The two runs lasted for 4,000,000 generations. The covarion (COV) model was used together with the GTR+G+I to accommodate for different substitution rates across sites (G + proportion of invariable sites (I)) and across sequences (COV). The covarion model included two parameters, sites being on > off and off > on. All phylogenetic analyses were done on the freely available Bioportal at University of Oslo http://www.bioportal.uio.no.

## Authors' contributions

JB collected the freshwater samples, generated the sequence data, performed the phylogenetic analyses, and wrote the manuscript. TR collected environmental samples and designed and tested the Telonemia specific primers. DK supervised and participated in the sample collection and manuscript writing. KSJ funded and coordinated the study and contributed to writing the manuscript. All authors have read and approved the final manuscript. KST designed the project, supervised the analyses and interpretation of the molecular phylogenies and participated in writing the manuscript.

## Supplementary Material

Additional file 1 **Supplementary table Description of sequences used in the phylogenetic analyses in **Figure 1. Sequences in bold are generated in this study. Click here for file
