# Supplementary material for: Telonemia-specific environmental 18S rDNA PCR reveals unknown diversity and multiple marine-freshwater colonizations
Source: BMC Microbiol. 2010 Jun 9;10:168. doi: 10.1186/1471-2180-10-168 (PMC2891722; doi:10.1186/1471-2180-10-168)
Supplement: Additional file 1 — Supplementary table Description of sequences used in the phylogenetic analyses in Figure 1. Sequences in bold are generated in this study. [file 1471-2180-10-168-S1.DOCX]

**Supplementary table:**

**Description of sequences used in the phylogenetic analyses in Figure 1.** Sequences in bold are generated in this study.

| Sample localization | Accession numbers | Clone numbers/species names/DGGE band |
| --- | --- | --- |
| Canada Basin, Arctic Ocean | DQ119997 | NW614.39 |
| Canada Basin, Arctic Ocean | DQ119996 | NW614.31 |
| Canada Basin, Arctic Ocean | DQ120001 | NW617.20 |
| Canada Basin, Arctic Ocean | DQ120000 | NW414.45 |
| Canada Basin, Arctic Ocean | DQ119998 | NW414.01 |
| Canada Basin, Arctic Ocean | DQ119999 | NW414.42 |
| English Channel, Roscoff, France | AJ564771 | *Telonema subtilis* (RCC404.5) |
| English Channel, Roscoff, France | AY295352 | *Telonema subtilis* (RCC358.7) |
| English Channel, Roscoff, France | AY295583 | RA010412.17 |
| English Channel, Roscoff, France | AY295375 | RA000412.136 |
| English Channel, Roscoff, France | AY295476 | RA000609.57 |
| English Channel, Roscoff, France | AY295470 | RA000609.49 |
| English Channel, Roscoff, France | AY295644 | RA010516.38 |
| English Channel, Roscoff, France | AY295498 | RA000907.26 |
| English Channel, Roscoff, France | AY295501 | RA000907.3 |
| English Channel, Roscoff, France | AY295513 | RA000907.47 |
| Helgoland, Germany | AJ965240 | He000427.29 |
| Oslo Fjord, Norway | AJ564773 | *Telonema antarcticum* |
| Mariager Fjord, Denmark | DQ103867 | M318B12 |
| Mariager Fjord, Denmark | DQ103827 | M218G12 |
| Xiamen Islands, China | DQ667668 | XMCF11 |
| Hawaii | EU499983 | dhot1a7 |
| Hawaii | EF695209 | hotp1h2 |
| Hawaii | EU500219 | hotxp4c7 |
| Hawaii | EU499984 | dhot1d9 |
| Western North Atlantic | DQ918262 | ENVP21819.00002 |
| Western North Atlantic | DQ917977 | ENVP10203.00063 |
| Western North Atlantic | DQ918437 | ENVP21819.00370 |
| Western North Atlantic | DQ918372 | ENVP21819.00243 |
| Western Pacific | EF539138 | MB04.38 |
| Sargasso Sea | AY665040 | SCM27C23 |
| Sargasso Sea | AY665043 | SCM27C46 |
| Sargasso Sea | AY665042 | SCM27C3 |
| Sargasso Sea | AY665041 | SCM27C12 |
| Sargasso Sea | AY665037 | SCM38C20 |
| Indian Ocean | AM418562 | IND33.54 |
| Indian Ocean | AM418563 | IND31.100 |
| **Indian Ocean** | **GQ365830** | **IND31.Telo.43** |
| **Indian Ocean** | **GQ365824** | **IND31.Telo.38** |
| **Indian Ocean** | **GQ365855** | **IND72.Telo.5** |
| **Indian Ocean** | **GQ365845** | **IND60.Telo.3** |
| **Indian Ocean** | **GQ365831** | **IND31.Telo.44** |
| **Indian Ocean** | **GQ365842** | **IND33.Telo.6** |
| **Indian Ocean** | **GQ365836** | **IND31.Telo.8** |
| **Indian Ocean** | **GQ365799** | **IND31.Telo.10** |
| **Indian Ocean** | **GQ365859** | **IND72.Telo.9** |
| **Indian Ocean** | **GQ365805** | **IND31.Telo.2** |
| **Indian Ocean** | **GQ365808** | **IND31.Telo.22** |
| **Indian Ocean** | **GQ365817** | **IND31.Telo.31** |
| **Indian Ocean** | **GQ365800** | **IND31.Telo.11** |
| **Indian Ocean** | **GQ365815** | **IND31.Telo.3** |
| **Indian Ocean** | **GQ365822** | **IND31.Telo.36** |
| **Indian Ocean** | **GQ365809** | **IND31.Telo.23** |
| **Indian Ocean** | **GQ365847** | **IND60.Telo.5** |
| **Indian Ocean** | **GQ365802** | **IND31.Telo.16** |
| **Indian Ocean** | **GQ365823** | **IND31.Telo.37** |
| **Indian Ocean** | **GQ365840** | **IND33.Telo.3** |
| **Indian Ocean** | **GQ365804** | **IND31.Telo.19** |
| **Indian Ocean** | **GQ365839** | **IND33.Telo.2** |
| Antarctica: Ellis Fjord, Vestfold Hills | DQ507401 | SMB_Euk_DGGE1 |
| **Antarctica** | **GU117661** | **Dhr41.Telo.4** |
| **Antarctica** | **GU117662** | **Dhr41.Telo.16** |
| **Antarctica** | **GU117665** | **Dhr41.Telo.15** |
| **Antarctica** | **GU117663** | **Dhr41.Telo.1** |
| **Antarctica** | **GU117664** | **Dhr41.Telo.11** |
| Arctic Ocean, Svalbard | DQ119993 | NOR26.38 |
| Arctic Ocean, Svalbard | DQ119995 | NOR46.11 |
| Arctic Ocean, Svalbard | DQ119994 | NOR26.35 |
| Arctic Ocean, Svalbard | DQ647533 | PD6.20 |
| Arctic Ocean, Svalbard | DQ647539 | AD6S.06 |
| **Arctic Ocean, Svalbard** | **GQ365768** | **AD6.Telo.4** |
| **Arctic Ocean, Svalbard** | **GQ365764** | **AD6.Telo.13** |
| **Arctic Ocean, Svalbard** | **GQ365767** | **AD6.Telo.20** |
| **Arctic Ocean, Svalbard** | **GQ365766** | **AD6.Telo.2** |
| **Arctic Ocean, Svalbard** | **GQ365765** | **AD6.Telo.19** |
| **Arctic Ocean, Svalbard** | **GQ365882** | **NOR46.Telo.23** |
| **Arctic Ocean, Svalbard** | **GQ365861** | **NOR26.Telo.10** |
| **Arctic Ocean, Svalbard** | **GQ365867** | **NOR26.Telo.9** |
| **Arctic Ocean, Svalbard** | **GQ365866** | **NOR26.Telo.7** |
| **Arctic Ocean, Svalbard** | **GQ365865** | **NOR26.Telo.6** |
| **Arctic Ocean, Svalbard** | **GQ365889** | **NOR46.Telo.3** |
| **Arctic Ocean, Svalbard** | **GQ365902** | **NOR46.Telo.41** |
| **Arctic Ocean, Svalbard** | **GQ365879** | **NOR46.Telo.20** |
| **Arctic Ocean, Svalbard** | **GQ365872** | **NOR46.Telo.14** |
| **Arctic Ocean, Svalbard** | **GQ365881** | **NOR46.Telo.22** |
| **Arctic Ocean, Svalbard** | **GQ365890** | **NOR46.Telo.30** |
| **Arctic Ocean, Svalbard** | **GQ365884** | **NOR46.Telo.25** |
| **Arctic Ocean, Svalbard** | **GQ365899** | **NOR46.Telo.39** |
| **Arctic Ocean, Svalbard** | **GQ365877** | **NOR46.Telo.19** |
| **Arctic Ocean, Svalbard** | **GQ365900** | **NOR46.Telo.4** |
| **Arctic Ocean, Svalbard** | **GQ365868** | **NOR46.Telo.1** |
| **Arctic Ocean, Svalbard** | **GQ365870** | **NOR46.Telo.12** |
| **Mediterranean Sea, Spain** | **GQ365776** | **BL040126.Telo.16** |
| **Mediterranean Sea, Spain** | **GQ365789** | **BL040126.Telo.31** |
| **Mediterranean Sea, Spain** | **GQ365769** | **BL040126.Telo.1** |
| **Mediterranean Sea, Spain** | **GQ365770** | **BL040126.Telo.10** |
| **Mediterranean Sea, Spain** | **GQ365798** | **BL040126.Telo.9** |
| **Mediterranean Sea, Spain** | **GQ365796** | **BL040126.Telo.6** |
| **Mediterranean Sea, Spain** | **GQ365781** | **BL040126.Telo.23** |
| **Mediterranean Sea, Spain** | **GQ365780** | **BL040126.Telo.22** |
| **Mediterranean Sea, Spain** | **GQ365788** | **BL040126.Telo.30** |
| **Mediterranean Sea, Spain** | **GQ365782** | **BL040126.Telo.24** |
| **Mediterranean Sea, Spain** | **GQ365794** | **BL040126.Telo.36** |
| **Mediterranean Sea, Spain** | **GQ365778** | **BL040126.Telo.18** |
| Mediterranean Sea, Spain | AJ564770 | BL010625.25 |
| Mediterranean Sea, Spain | AY426930 | BL010625.26 |
| Framvaren Fjord, Norway | EF526966 | SA1_2A10 |
| Framvaren Fjord, Norway | EF526892 | NA2_1F1 |
| Framvaren Fjord, Norway | EF526897 | SA1_4A6 |
| Framvaren Fjord, Norway | EF526718 | NA1_2B9 |
| Framvaren Fjord, Norway | EF526860 | NA1_1D3 |
| **Lake Lutvann sediment 20 m, Norway** | **GU117669** | **Lut.Sed.20.9** |
| **Lake Lutvann sediment 20 m, Norway** | **GU117668** | **Lut.Sed.20.8** |
| **Lake Lutvann sediment 50 m, Norway** | **GU117666** | **Lut.Sed.50.3** |
| **Lake Lutvann sediment 5 m, Norway** | **GU117671** | **Lut.Sed.5.5** |
| **Lake Lutvann sediment 5 m, Norway** | **GU117670** | **Lut.Sed.5.2** |
| **Lake Lutvann sediment 20 m, Norway** | **GU117667** | **Lut.Sed.20.2** |
| **Lake Sværsvann, Norway** | **GU117690** | **Svv.Telo.1** |
| **Lake Sværsvann, Norway** | **GU117691** | **Svv.Telo.3** |
| **Lake Sværsvann, Norway** | **GU117693** | **Svv.Telo.10** |
| **Lake Lutvann, Norway** | **GU117679** | **Lut.Telo.16** |
| **Lake Lutvann, Norway** | **GU117672** | **Lut.Telo.23** |
| **Lake Pollen, Norway** | **GU117689** | **Pol.Telo.10** |
| **Lake Pollen, Norway** | **GU117687** | **Pol.Telo.7** |
| **Lake Lutvann, Norway** | **GU117678** | **Lut.Telo.9** |
| **Lake Lutvann, Norway** | **GU117673** | **Lut.Telo.21** |
| **Lake Pollen, Norway** | **GU117683** | **Pol.Telo.1** |
| **Lake Lutvann, Norway** | **GU117677** | **Lut.Telo.12** |
| **Lake Lutvann, Norway** | **GU117675** | **Lut.Telo.24** |
| **Lake Pollen, Norway** | **GU117688** | **Pol.Telo.9** |
| **Lake Pollen, Norway** | **GU117684** | **Pol.Telo.2** |
| **Lake Lutvann, Norway** | **GU117682** | **Lut.Telo.15** |
| **Lake Lutvann, Norway** | **GU117674** | **Lut.Telo.19** |
| **Lake Pollen, Norway** | **GU117685** | **Pol.Telo.3** |
| **Lake Lutvann, Norway** | **GU117681** | **Lut.Telo.13** |
| **Lake Lutvann, Norway** | **GU117680** | **Lut.Telo.2** |
| **Lake Lutvann, Norway** | **GU117676** | **Lut.Telo.20** |
| **Lake Sværsvann, Norway** | **GU117692** | **Svv.Telo.4** |
| **Lake Pollen, Norway** | **GU117686** | **Pol.Telo.4** |
| Lake Bourget, France | EF196682 | BA4 |
| Lake Pavin, France | EU162631 | PTG4SP2005 |
| Glacial sample, Spitsbergen, Svalbard | EU371359 | NPK97_27 |
| Glacial sample, Spitsbergen, Svalbard | EU371360 | NPK97_25 |
| Glacial sample, Spitsbergen, Svalbard | EU371378 | NPK97_54 |
| Glacial sample, Spitsbergen, Svalbard | EU371358 | NPK97_7 |
| Glacial sample, Spitsbergen, Svalbard | EU371379 | NPK97_119 |
| Arctic Ocean | EU371188 | NPK2_190 |
| Arctic Ocean | EU371332 | NPK60_36 |
| Drinkwater Lake, Spitsbergen, Svalbard | EU078264 | DL-2-2 |
| Bayelva River, Spitsbergen, Svalbard | EU078256 | B-2-8 |
| Hyperhaline lake, Chile | AM179808 | DGGE band 20 |
| Kings Bay, Spitsbergen, Svalbard | EU050980 | SS1 E 01 20 |
| Kings Bay, Spitsbergen, Svalbard | EU050979 | SS1 E 01 55 |
|  | L28811 | Chilomonas paramecium |
|  | X57162 | Guillardia theta |
|  | AB231617 | Katablepharis japonica |
|  | AJ246269 | Prymnesium parvum |
|  | AB183618 | Emiliania sp. |
|  | L34669 | Pavlova salina |
